# Traumatic Brain Injury and Axonal Damage in the Pathogenesis of Frontotemporal Dementia: A Narrative Review

**DOI:** 10.7759/cureus.105586

**Published:** 2026-03-21

**Authors:** Saad A Farooqui, Fozia Farooqui

**Affiliations:** 1 Department of Emergency Medicine, Saint Mary Medical Center, Langhorne, USA; 2 Department of Neuroscience, Temple University, Philadelphia, USA; 3 Department of Hospital Medicine, Temple University Hospital, Philadelphia, USA

**Keywords:** amyloid beta plaque, frontotemporal dementia (ftd), role of neurons, traumatic axonal injury, traumatic brain injury (tbi)

## Abstract

Traumatic brain injury (TBI) has emerged as a critical environmental risk factor for the development of frontotemporal dementia (FTD), a neurodegenerative disorder characterized by progressive declines in executive function, behavior, and language. While the acute symptoms of head trauma often resolve, the long-term neurological consequences can include a significant increase in FTD susceptibility. A comprehensive search of electronic databases, including PubMed and Google Scholar, was conducted to identify relevant peer-reviewed studies published through 2026. This review examines the complex pathological mechanisms linking blunt force trauma to frontotemporal degeneration, specifically focusing on the role of traumatic axonal injury (TAI) and the subsequent accumulation of beta-amyloid plaques alongside related FTD-associated proteinopathies.

## Introduction and background

A traumatic brain injury (TBI) occurs when an individual suffers an injury to the head from an external force, such as blunt trauma or a fall, often resulting in acute and chronic neurological deficits that range from minor headaches to death [1]. Epidemiologic studies estimate that millions of people experience TBI annually worldwide, with many facing long-term health consequences [2]. While the immediate effects of TBI may subside, current research suggests that individuals remain susceptible to developing neurodegenerative conditions later in life, including frontotemporal dementia (FTD) [1,3]. FTD is characterized by psychological and behavioral deficits likely caused by protein damage induced by trauma [4]. While FTD is classically associated with tau and transactive response (TAR) DNA-binding protein 43 (TDP-43) proteinopathies, emerging research has also explored the potential role of amyloid-mediated pathways. One proposed mechanism linking TBI to FTD involves traumatic axonal injury (TAI), which increases amyloid plaque buildup in the brain [5,6]. While FTD is classically defined by tau and TDP-43 proteinopathies, emerging research suggests that beta-amyloid accumulation can occur as a co-pathology in a subset of patients, potentially serving as an early indicator of the neurodegenerative onset triggered by trauma [7,4,8].

To investigate the correlation between TBI and FTD, Asken et al. conducted a study analyzing participants from FTD memory care facilities and assessing factors such as duration of loss of consciousness (LOC), method of injury, frequency of TBI, and age at injury [1]. The researchers found that the frequency of TBI with LOC had a negative linear correlation with the onset of FTD symptoms. Patients with no history of TBI experienced an average symptom onset age of 63 years, whereas patients with two or more TBIs experienced onset at an average age of 55 years [1]. Additionally, the study found that healthy controls played an average of two years of American football, while the FTD sample played an average of five years. These findings suggest that repeated TBI and repetitive head trauma from contact sports are correlated with the development of FTD and may serve as contributing factors to its pathogenesis [1,9]. While these studies may indicate a significant association, current literature primarily supports a correlational rather than a purely causal link between TBI and subsequent dementia.

TBIs may lead to marked increases in amyloid plaques. TBIs are thought to increase the risk of neurodegenerative disease in part due to the TAI that occurs subsequent to the damage [5,10]. The TAI produces an increase in beta-amyloid protein buildup, called amyloid plaques, which, in turn, can produce further neurotoxicity and degeneration. Scott et al. sought to analyze the connection between TBIs and amyloid plaques [5]. Twenty-eight participants, nine with TBI, ten with Alzheimer's disease (AD), and nine controls, were assessed based on beta-amyloid buildup. The results of the study showed a positive correlation between TBI and TAI in TBI patients compared to the AD and control groups. Further analysis showed that carbon-11 Pittsburgh compound B non-displaceable binding potential (C-PiB BPND) increased by up to six-fold compared with the control and AD groups [5,11]. The large elevation of C-PiB BPND in TBI patients compared to control patients suggests amyloid plaque buildup is correlated with TAI resulting from TBI.

Evidence suggests that amyloid pathology may serve as an associated pathological feature in certain cases of FTD [7,12]. In FTD, beta-amyloid proteins clump in the frontal and temporal regions of the brain. This clumping causes loss of function, which accounts for the onset of FTD. The loss of function often includes neuronal death, which leads to coordination imbalance, language deficits, and abnormalities with executive function. It is important to note that while this review focuses on amyloid accumulation, the broader pathogenesis of FTD involves complex interactions between various protein aggregates, including tau and TDP-43.

Tan et al. used statistical imaging analysis to examine amyloid ligands and the correlation between FTD and beta-amyloid proteins in post-mortem brains [7]. The results of this experiment showed a strong presence of beta-amyloid proteins in the white matter of FTD brains, in contrast to an insignificant correlation of beta-amyloid in the pons, cerebellum, and medulla [7,13]. The positive correlation suggests that the increase in beta-amyloid protein in the white matter of the cortex may cause FTD.

Based on the aforementioned studies, patients with TBIs develop an increase in beta-amyloid plaques that may lead to FTD. The study by Asken et al. analyzes the correlation between TBI and FTD through frequency assessment in TBI and control patients [1]. The study by Scott et al. observes increases in beta-amyloid plaque status post-TBI using various imaging modalities [5]. The study by Tan et al. examines beta-amyloid protein buildup in the white matter of FTD patients [7]. This review synthesizes evidence that individuals suffering TBIs have a higher likelihood of developing FTD due to an increase in beta-amyloid plaques resulting from trauma.

## Review

Methods

A comprehensive search of electronic databases, including PubMed and Google Scholar, was conducted to identify peer-reviewed studies published through 2025 focusing on the intersection of TBI, amyloid pathology, and FTD. Studies were selected based on their relevance to traumatic axonal injury and proteinopathy, with a preference for those providing quantitative neuroimaging or histopathological data.

Results

Epidemiological Evidence

In the study conducted by Asken et al., the researchers sought to determine whether TBIs and repetitive head impacts (RHIs) are associated with a higher risk of FTD [1]. To determine the correlation between TBI and FTD, Asken et al. recruited 264 participants. Of these, 132 were FTD or primary progressive aphasia (PPA) patients with a mean age of 68.9 ± 8.1 years, and 65% were male. These patients were recruited from three University of California, San Francisco memory clinics [1]. They were age- and sex-matched with 132 controls with a mean age of 73.4 ± 7.6 years who were unaffected by FTD or PPA. Inclusion criteria for the experimental group required a diagnosis of behavioral variant FTD (bvFTD), several forms of PPA, or cortical basal syndrome (CBS). No exclusion criteria were specified, and the duration of the study was not provided.

To determine whether TBIs negatively impact FTD, the researchers first used the Ohio State TBI Identification and Boston University Head Impact Exposure Assessment to analyze the frequency and severity of TBIs in both groups. The onset of symptoms was obtained based on subjective reports from the patient or their study partner. Comprehensive neurophysiological assessments, including the 15-item Boston Naming Test and the animals/one-minute test, were used to determine clinical outcomes. Results were analyzed using analysis of variance (ANOVA), stepwise trends, neuropsychiatric symptoms (NPI-Q total score), and composite domain z-scores, with p < 0.05 considered significant.

Referring to the results of this study, patients with zero TBIs had an FTD onset age of 62.1 ± 8.1 years, those with one TBI had an onset age of 59.9 ± 6.9 years, and those with two or more TBIs had an onset age of 57.3 ± 8.4 years (p = 0.03) [1]. This suggests that increased TBIs are associated with earlier onset of FTD. The results further demonstrate that patients who played American football and experienced RHI also followed a negative trend with FTD. Zero years of football correlated with an FTD onset age of 62.2 ± 8.7 years, one to four years correlated with an onset age of 59.7 ± 7.0 years, and five or more years correlated with an onset age of 55.9 ± 6.3 years (p = 0.009). These findings suggest that repetitive blows to the head correlate with an earlier onset of FTD. The results, based on the z-score memory test, show a negative correlation between memory performance and amount of American football play (p = 0.02), indicating that increased head trauma may contribute to memory deficits [1,9].

Neuroimaging and Pathological Evidence

Scott et al. conducted a study to investigate beta-amyloid pathology status following TBI [5]. To assess whether TBIs influenced amyloid pathology, the researchers recruited 28 participants. The experimental group consisted of nine participants with a mean age of 44.1 ± 4.9 years who had experienced moderate to severe TBIs at least 11 months prior to recruitment, as classified by the Mayo Criteria. Ten participants with AD had a mean age of 67.3 ± 4.5 years. Nine participants served as age-matched controls with a mean age of 62.3 ± 4.3 years, comprising three total control groups.

The experimental group was assessed using carbon-11 Pittsburgh compound B positron emission tomography (C-PiB-PET), structural T1-weighted magnetic resonance imaging (T1-MRI), diffusion tensor imaging (DTI), and neurophysiological examination. The AD group underwent C-PiB-PET and structural MRI. Control group one underwent C-PiB-PET and structural MRI. Control group two underwent neurophysiological assessment. Control group three underwent structural MRI and DTI. Inclusion and exclusion criteria were unclear, and the length of the assessment was not specified.

Results were analyzed using t tests, Mini-Mental State Exams, Mann-Whitney U tests, nonparametric permutation tests, region of interest (ROI) analysis, and ANOVA. A p-value of less than 0.001 was considered significant, and the false discovery rate was calculated with q = 0.05. The results demonstrate a significant increase in beta-amyloid plaques marked by C-PiB for AD and TBI patients compared to healthy controls (p < 0.001), though raw values were not provided. This suggests that TBIs are linked to increased amyloid pathology secondary to axonal damage, consistent with patterns observed in AD [5,11]. The research also found a positive correlation between duration since TBI and C-PiB binding in the posterior cingulate cortex (R = 0.767, p = 0.021). This indicates that TBIs are associated with chronic neurological beta-amyloid plaque buildup, as C-PiB serves as a marker for amyloid plaques in the brain. However, because C-PiB imagery is qualitative, the exact increase remains difficult to quantify.

Neuropathological Synthesis

In the study by Tan et al., researchers analyzed beta-amyloid pathology in FTD patients to determine a correlation [7]. To assess whether increases in beta-amyloid pathology lead to a higher likelihood of FTD, Tan et al. analyzed 134 post-mortem brains. Ninety-eight of these brains were autopsy-confirmed FTD brains meeting inclusion criteria, and 36 were control brains. These samples were obtained from the Sydney Brain Bank and the Cambridge Brain Bank, ethically following local ethics committee guidelines. Exclusion criteria included excessive cortical neuritic plaque formation based on the Braak NFT score of two or higher. Researchers then conducted genetic mutation analysis and Pittsburgh compound B positron emission tomography (PiB-PET) analysis to assess beta-amyloid formation.

Statistical analysis was performed using Statistical Package for the Social Sciences (SPSS) (IBM Corp., Armonk, New York, USA), with ANOVA and Bonferroni-corrected post hoc tests (p < 0.05). Pearson's correlation coefficient was also used with a significance threshold of p < 0.03. Tan et al. reported that the volume fraction of beta-amyloid in the frontal cortex of bvFTD brains was 2.6 ± 4.0, compared to 1.5 ± 2.0 in age-matched controls and 16.9 ± 10.0 in age-matched AD (p < 0.005 compared to AD) [7]. The same volume in the temporal cortex of bvFTD brains was 3.5 ± 5.8, compared to 0.6 ± 0.7 in age-matched controls and 14.3 ± 6.7 in AD (p < 0.005 compared to AD). These findings suggest that amyloid pathology is more prevalent in FTD brains than in controls, though less prevalent than in AD brains [7,13].

Discussion

Synthesis of Findings

TBIs demonstrate a positive correlation with beta-amyloid pathology, and increases in amyloid pathology correlate with increased FTD symptoms. Asken et al. found that increased TBIs lead to memory deficits as measured by several neurological assessments [1]. Scott et al. documented the presence of amyloid pathology secondary to TBIs compared to healthy controls [5,11]. Tan et al. established a correlation between amyloid plaque buildup and FTD diagnosis [7]. Together, these findings suggest that increased amyloid pathology secondary to TBI may contribute to the development of FTD.

The collective findings suggest a biological sequence linking head trauma to frontotemporal dementia. The mechanical force of a TBI initiates traumatic axonal injury, stretching and tearing neuronal fibers. This axonal damage acts as a critical intermediate step by disrupting normal transport and clearance mechanisms. Consequently, proteins such as beta-amyloid begin to accumulate in the brain rather than being cleared [6,10]. The connection between TBI and neurodegeneration is therefore a continuous pathological process that begins at the moment of impact. This sequence suggests that axonal integrity is a primary determinant of long-term proteinopathy risk.

The accumulation of beta-amyloid plaques following axonal injury provides a direct link to the pathology observed in FTD patients. Studies examining post-mortem FTD brains confirm that these amyloid plaques are present in significant quantities, particularly in the frontal and temporal regions that govern behavior and language [7,8]. This convergence of findings is important. It demonstrates that the same type of pathology seen after a TBI is also found in the brains of individuals who suffered from FTD. This suggests a progressive timeline where an initial traumatic event sets the stage for a decades-long pathological process. While research suggests that trauma-induced axonal injury facilitates amyloid plaque formation and subsequent functional decline, this proposed sequence likely represents one of several interacting pathways, including tau aggregation and neuroinflammation, that collectively contribute to the complex pathogenesis of frontotemporal dementia [12,13]. The dose-response relationship observed in the TBI study, where more head injuries led to earlier symptom onset, further supports this timeline. More trauma means more axonal injury, which means a greater burden of plaque accumulation and ultimately faster progression to clinical symptoms.

The clinical data regarding repetitive head impacts from sports such as American football adds a crucial layer to this understanding. These athletes do not always experience TBIs with loss of consciousness in the traditional sense. Instead, they endure hundreds or thousands of subconcussive hits over years of play. Each of these impacts may cause microscopic axonal damage that is not severe enough to produce immediate symptoms [9,14]. However, the cumulative effect of this damage over time appears to mirror the pathology seen in patients with documented TBIs. The athletes in the FTD sample played significantly more football than the healthy controls, and they experienced earlier cognitive decline and memory deficits [1,9]. This reinforces the idea that the key factor is not always a single dramatic injury, but the total burden of axonal trauma sustained over a lifetime. Whether from a few severe injuries or many years of repetitive impact, the resulting axonal injury and subsequent amyloid pathology appear to follow a similar pathway toward the development of frontotemporal dementia.

Limitations and Future Directions

Asken et al. aimed to determine if TBIs and repetitive head impacts increased the risk of FTD based on analysis of symptoms [1]. The study found that increased American football play led to greater susceptibility to TBIs and RHIs, which correlated with greater susceptibility to FTD. However, the study compared the FTD patient group with a sex-matched control group that had an age difference of 4.5 years. This is significant because FTD prevalence increases with age [15]. Since the control group was 4.5 years older than the experimental group, the control group was more susceptible to developing FTD. This undermines the accuracy of the control group by introducing an age-related variable. The experimental group had less age-related risk, potentially skewing results. Future experiments must age-match healthy controls to FTD patients more accurately to produce valid results.

Another limitation involves the sex distribution in the study. Sixty-five percent of FTD patients were male, which does not accurately represent sex differences in FTD development. In a meta-analysis conducted by Curtis et al., two-thirds of FTD variants showed no sex difference, while analysis of the third variant showed that females had a higher prevalence of developing FTD [16]. Future experiments should include an equal or near-equal ratio of males and females that is representative of true sex differences in FTD development. A third limitation concerns the type of TBI analyzed. This study primarily focused on football-related injuries, which narrows the scope of results. TBI is a general term, and differences in impact mechanisms may yield different neurological outcomes. To address this limitation, researchers should seek a broader range of individuals with TBIs from diverse causes.

Scott et al. analyzed beta-amyloid pathology after TBI [5]. Blunt force injuries causing TBIs lead to acute or chronic anatomical changes in the brain. These changes include axonal damage, which in some cases leads to uncontrolled beta-amyloid plaque buildup. A low sample size, with only nine TBI patients, represents a significant limitation. Small sample sizes can lead to misrepresentation of amyloid pathology in TBI populations. A more robust study should include approximately 50 TBI patients with their respective controls. Another limitation involves the variable duration between TBI and patient assessment. Researchers recruited TBI patients from 11 months to 17 years after injury. Amyloid pathology is age-related and develops over time. A patient with a TBI 11 months prior may have less plaque aggregation compared to a patient with a TBI 17 years prior, yet this variable was not adequately controlled. Future studies should analyze patients with TBIs occurring within a narrower timeframe, such as 15 ± 1 years ago, to ensure adequate and comparable plaque aggregation.

A third limitation involves the neurophysiological assessments used in the study. Subjective reports and memory question responses are not quantifiable and may vary inconsistently between individuals. Navigating this limitation is difficult because neurophysiological assessments are inherently subjective. Future experiments should incorporate quantitative or objectively verifiable measures where possible to improve reliability.

Tan et al. used neuroimaging to analyze beta-amyloid pathology in FTD patients [7]. While previous research has implicated tau and TAR DNA-binding protein 43 (TDP-43) proteins in FTD, this study demonstrates a correlation between beta-amyloid plaques and FTD. This finding provides new insight into potential disease mechanisms and is significant for understanding FTD etiology. However, it remains unclear whether beta-amyloid plaques are a primary cause of FTD or simply a consequence of age-related degeneration. Future studies should investigate whether extensive plaque formation directly causes FTD symptoms. This could be accomplished through C-PiB imaging in living patients under age 50, where FTD symptoms could be analyzed in relation to imaging findings. Patients under 50 are less susceptible to age-related degeneration, so a causative relationship might be more clearly observable.

Another limitation involves the geographic origin of brain samples. Samples were obtained from two brain banks, one in Sydney, Australia and the other in Cambridge, United Kingdom. Treatment approaches for FTD may vary by location, potentially resulting in different healthcare outcomes and pathological presentations. This limits the generalizability of findings to all FTD patients. Future experiments should utilize global brain banks, such as the Concussion Legacy Foundation, which focuses on brain trauma but also houses FTD specimens relevant to both TBI and amyloid pathology. This would diversify the sample and improve representativeness. Additionally, researchers sectioned the left frontal cortex but not the right frontal cortex when analyzing the topographical distribution of beta-amyloid pathology. This omits half of the frontal cortex, leaving hemisphere differences in amyloid pathology unexamined [16]. Future experiments should section both hemispheres equally to enable accurate identification of potential hemispheric differences.

In a study by Bibl et al., researchers analyzed differences in beta-amyloid 42 levels in FTD and AD patients [17]. They found that amyloid pathology was unaffected and normal in all 17 FTD patients and 20 non-demented patients. In contrast, amyloid pathology was abnormal in all 22 AD patients. These findings conflict with those of Tan et al., who reported increased amyloid pathology indicative of FTD symptoms [7]. The Bibl study suggests that amyloid peptides are not necessary for FTD development but rather are specific to AD. This would also conflict with the findings of Scott et al., as it implies that amyloid pathology following TBI may be insignificant in relation to FTD [5,17]. Further research is needed to resolve these discrepancies and clarify the role of amyloid in FTD pathogenesis.

The studies reviewed in this comprehensive analysis provide compelling evidence for a correlation between traumatic brain injury and the subsequent development of frontotemporal dementia. The research conducted by Asken and colleagues demonstrates a clear association between repetitive head impacts and earlier onset of FTD symptoms, particularly in individuals with a history of multiple TBIs or participation in contact sports [1]. This epidemiological evidence is supported by the work of Scott and colleagues, who identified a potential biological mechanism for this correlation. Their findings indicate that TBIs can lead to traumatic axonal injury, which in turn is associated with a significant increase in beta-amyloid plaque buildup in the brain [5]. The presence of this amyloid pathology is particularly relevant given the research by Tan and colleagues, which confirmed a strong correlation between elevated beta-amyloid proteins in the cortical white matter and the diagnosis of FTD [7].

Taken together, these studies suggest a sequential pathway in which TBI-induced axonal damage triggers the accumulation of beta-amyloid plaques, and this pathological change may contribute to the onset of frontotemporal dementia. While conflicting findings from Bibl and colleagues regarding the role of amyloid peptides in FTD warrant further investigation, the preponderance of evidence from the primary studies supports the proposed link between head trauma, amyloid pathology, and FTD [17]. Future research should aim to address the limitations outlined in this review, including the need for larger sample sizes, more diverse participant populations, and stringent age-matching in control groups. A more precise understanding of this relationship is essential for developing improved diagnostic criteria and potential therapeutic targets for individuals at risk for FTD following traumatic brain injury.

Emerging Insights into Traumatic Brain Injury (TBI)-Induced Neurodegeneration

Recent epidemiological research has strengthened the link between traumatic brain injury and various forms of dementia. A large meta-analysis examining over one hundred studies found that TBI was associated with significantly increased odds of developing all-cause dementia, Alzheimer's disease, vascular dementia, and frontotemporal dementia [18]. The strongest association was observed for frontotemporal lobar degeneration, with an odds ratio approaching four. This suggests that the relationship between head trauma and FTD may be particularly robust compared to other neurodegenerative conditions. The variation in subtype-specific associations indicates that different mechanisms may underlie the connection between TBI and distinct forms of dementia.

The search for reliable biomarkers has advanced considerably in recent years. A living systematic review and meta-analysis evaluated six core blood-based biomarkers for predicting outcomes after TBI [19]. The study found that glial fibrillary acidic protein and ubiquitin C-terminal hydrolase-L1 showed strong associations with both in-hospital and six-month mortality. The clinical utility of these markers is particularly relevant to FTD research, as they may help identify the early window of astrogliosis and neuronal injury that precedes frontotemporal symptom onset. However, the authors noted serious problems in the design and analysis of many studies, concluding that there remains little evidence that any of these markers are ready for clinical implementation. This cautionary note highlights the gap between research findings and clinical application.

Neuroimaging studies have begun to reveal how TBI alters brain function in ways that may accelerate pathological aging. A coordinate-based meta-analysis of fMRI studies examined functional connectivity changes after TBI across 76 studies with over 5,000 participants [20]. The analysis found that mild TBI most frequently affected the default mode, ventral attention, and somatomotor networks, while moderate to severe TBI involved the frontoparietal and attention networks. These functional disruptions in the frontoparietal networks align with the executive and behavioral deficits hallmark to FTD, suggesting a potential imaging-based link between injury location and dementia subtype. Although consistent patterns emerged, corrected statistical significance was not achieved, highlighting the need for larger harmonized datasets and standardized analysis pipelines in future research.

Longitudinal studies have provided further evidence that TBI creates distinct patterns of brain change. Research examining participants with mild cognitive impairment found that those with prior TBI displayed greater declines in local connectivity throughout both temporal and frontal lobes over a two-year period [21]. The TBI group also demonstrated unique increases in prefrontal cortex global connectivity, suggesting that these individuals may represent a distinct diagnostic category. This finding raises the possibility that mild cognitive impairment with a history of TBI may follow a different trajectory than cognitive impairment without head trauma.

Blood biomarker research has also explored how TBI modifies the association between protein levels and dementia risk. A community-based study found that TBI events alter the relationships between midlife and late-life plasma biomarkers and subsequent dementia [22]. Evidence of interaction by TBI status was observed for Alzheimer's-related biomarkers as well as for markers of neuronal injury and astrogliosis. This suggests that the pathological processes underlying TBI-related dementia are heterogeneous and that associations between biomarkers and outcomes may be bidirectional rather than unidirectional.

Emerging therapeutic strategies have focused on the earliest stages of post-injury pathology. Researchers have developed recombinant antibody fragments called nanobodies that specifically target toxic oligomeric protein variants without binding to monomeric or fibrillar forms [23]. These nanobodies can detect protein variants in blood and cerebrospinal fluid and may prove useful for diagnosing, staging, and treating neurodegenerative diseases and TBI. Their high specificity minimizes unwanted side effects and could provide safer long-term therapy by targeting an early cause rather than treating symptoms.

Another promising avenue involves restoring the brain's natural drainage system after injury. Research has shown that even a single mild TBI can disrupt the brain's lymphatic vessels, which help clear waste products and support immune function [24]. This disruption leads to the accumulation of harmful tau protein and widespread neurodegeneration. Given that tauopathy is a primary pathological driver of FTD, restoring lymphatic clearance represents a targeted strategy for preventing the protein aggregation that characterizes frontotemporal lobar degeneration. Importantly, intervention within 24 hours of injury using a lymphatic growth factor restored vessel function and prevented tau accumulation in laboratory studies. While far from ready for human use, this approach suggests a potential strategy for reducing long-term neurological consequences of head injuries.

Advances in neuroimaging have also improved understanding of post-TBI pathology. Positron emission tomography tracers can now evaluate alterations in metabolism, amyloid deposition, tau accumulation, neuroinflammation, and neurotransmitter systems after TBI [25]. These molecular imaging tools provide critical insights into pathophysiological processes, including disrupted glucose metabolism and chronic inflammatory responses. Integrating PET imaging into TBI diagnosis and longitudinal monitoring may help guide future therapeutic strategies, though challenges remain in standardizing these approaches for clinical use.

The neurovascular unit has emerged as a key player in both TBI and neurodegenerative disease. The blood-brain barrier regulates exchange between blood and the central nervous system, maintaining stability for proper brain function [26]. TBI and Alzheimer's disease share several histopathological hallmarks, including beta-amyloid aggregation, tau hyperphosphorylation, and plasma protein infiltration. A proposed shared cascade centered on the neurovascular unit suggests that increased blood-brain barrier permeability induces mechanisms leading to neuronal loss and cognitive dysfunction in both conditions. In the context of FTD, this neurovascular breakdown may be a critical precursor to the selective vulnerability of the frontal and temporal lobes following impact. This hypothesis offers a unifying framework for understanding how head trauma might trigger processes that ultimately result in dementia.

Finally, direct measurements of amyloid beta in the living brain after injury have revealed dynamic changes that correlate with patient status. Researchers collecting interstitial fluid from patients with severe TBI found that amyloid beta levels decreased as the neurological condition worsened and increased as patients improved [27]. The same dynamic relationship was not found in ventricular cerebrospinal fluid, raising questions about what different fluid compartments actually measure. This work demonstrates that processes occurring in the brain after injury may not be detected by standard fluid measurements, complicating efforts to understand the relationship between acute changes and long-term outcomes. The researchers noted that amyloid plaques have been shown to form rapidly over days in some people following head trauma, suggesting that some of the amyloid beta released during injury can come out of solution to form the plaques that characterize later neurodegeneration.

## Conclusions

The studies reviewed in this comprehensive analysis provide compelling evidence for a correlation between traumatic brain injury and the subsequent development of frontotemporal dementia. Research demonstrates a clear association between repetitive head impacts and an earlier onset of FTD symptoms, particularly in individuals with a history of multiple TBIs or participation in contact sports. This epidemiological evidence is supported by studies identifying a potential biological mechanism for this correlation, as findings indicate that TBIs can lead to traumatic axonal injury, which is associated with a significant increase in beta-amyloid plaque buildup in the brain. The presence of this amyloid pathology is particularly relevant given research confirming a strong correlation between elevated beta-amyloid proteins in the cortical white matter and the diagnosis of FTD. While this review focuses on the amyloid-mediated pathway, it is important to recognize that FTD is a heterogeneous disorder primarily defined by tau or TDP-43 proteinopathies. Taken together, these studies suggest a sequential pathway in which TBI-induced axonal damage triggers the accumulation of beta-amyloid plaques, and this pathological change may contribute to the onset of FTD. However, it must be emphasized that current evidence remains primarily associative, and further research is required to definitively establish causality. While conflicting findings regarding the role of amyloid peptides in FTD warrant further investigation, the preponderance of evidence from the primary studies supports the proposed link between head trauma, amyloid pathology, and FTD. Future research should aim to address the limitations outlined in this review, including the need for larger sample sizes, more diverse participant populations, and stringent age matching in control groups, as a more precise understanding of this relationship is essential for developing improved diagnostic criteria and potential therapeutic targets for individuals at risk for FTD following a traumatic brain injury.
